# Re-envisioning Kangaroo Mother Care Implementation Through a Socioecological Model: Lessons From Malawi

**DOI:** 10.9745/GHSP-D-21-00727

**Published:** 2022-08-30

**Authors:** Megan M. Lydon, Victoria Lwesha, Dyson Likomwa, Lydia Chimtembo, Tanya Guenther, Monica Longwe

**Affiliations:** a FHI 360, Durham NC, USA.; b Save the Children, Lilongwe, Malawi.; c Save the Children, Maputo, Mozambique.; d World Relief, Juba, South Sudan.; e Save the Children, Washington, DC, USA.; f Aurum Institute, Johannesburg, South Africa.

## Abstract

**Introduction:**

Kangaroo mother care (KMC) is a critical strategy to care for preterm and low birth weight infants in resource-limited settings. Despite evidence of its effectiveness and low cost, coverage has remained low, largely due to sociocultural barriers. We aimed to better understand social norms and community perceptions of preterm infants and KMC (facility-initiated and community-continued) in Malawi, a country with a high preterm birth rate, to inform a pilot social and behavior change program.

**Methods:**

In this qualitative study, we conducted 11 focus group discussions and 20 in-depth interviews. Participants were identified through purposive and snowball sampling and included pregnant women, parents engaged in KMC, health workers, community members, and religious leaders. Audio recordings were transcribed and translated into English. An inductive thematic analysis was applied.

**Results:**

Our analysis revealed 3 key injunctive norms influencing KMC engagement in this setting: (1) a perception that although preterm infants are abnormal, they should still be cared for, (2) an understanding that men should provide for their families while women should care for their families, and (3) families and communities should support one another. These norms operated at the community, family, household, and individual levels. Community members enforced social norms but also offered social support. Family members supported KMC parents as well. Both KMC parents actively engaged in the practice; however, their interest in and ability to manage its demands were influenced by the prevailing social norms, their economic situation, family obligations, and the mother’s health status.

**Conclusion:**

KMC efforts can be strengthened by incorporating context-specific actions to bolster social norms that align with KMC and shift those that discourage it. Activities must include a focus on the community and family levels to engender meaningful change at the household level.

## Abstract (precis)

Successful kangaroo mother care (KMC) efforts must understand and address social norms that influence this practice. The current study offers a model for how to connect social norms analysis to specific actions to improve KMC implementation.

## Teaser

**Key Findings:**

- This analysis identified 3 major injunctive norms that influence behavior at the community, family, household, and individual levels, and have both positive and negative effects on parent engagement with kangaroo mother care (KMC) in Malawi.
- Proposed areas for intervention to promote KMC include (1) emphasizing the value of life and working to shift community attitudes about preterm infants, (2) encouraging family and community support systems, (3) highlighting the discrepancy between perceived and actual male KMC involvement, (4) sharing strategies that support collaborative participation in KMC among both parents, and (5) developing or strengthening efforts to provide economic relief to KMC parents.

**Key Implications:**

- Because KMC requires significant caregiver involvement, a well-informed, context-specific social behavior change strategy is critical to increasing uptake and continuation.
- Successful promotion efforts will build upon social norms that support KMC and aim to shift those that limit it, with actions focusing on multiple social levels.

## INTRODUCTION

Kangaroo mother care (KMC) has been widely endorsed as an essential strategy to care for preterm and low birth weight (LBW) infants in resource-limited settings.^1^^–^^3^ KMC involves early, continuous, and prolonged skin-to-skin care, exclusive breastfeeding, and early discharge from the health facility with close at-home follow-up.^4^ Evidence shows that KMC effectively reduces the mortality of stabilized preterm and LBW infants by 40%.^5^ Recent research also indicates that both community-initiated and immediate KMC are safe and effective, reducing the risk of death among LBW newborns by 30% and 25%, respectively.^6^^,^^7^ KMC has been associated with a reduction in sepsis and an increase in breastfeeding.^5^ Furthermore, KMC is a low-cost, low-technology intervention that can be implemented across a range of settings.^8^

While KMC offers notable benefits, global uptake remains low.^2^^,^^8^^,^^9^ Several studies have highlighted barriers to KMC that help explain this.^9^^–^^12^ Health systems issues exist, including a lack of dedicated space, low levels of provider knowledge or motivation, and inadequate staff time to support parents with KMC.^10^^,^^12^^,^^13^ Social norms and beliefs, including negative cultural perceptions of preterm and LBW infants, have also led to poor KMC uptake. In some cultures, KMC clashes with traditional newborn care practices.^9^^,^^11^ Numerous studies have underscored the role of gender norms with respect to KMC engagement.^9^^,^^11^^,^^14^ Parent experiences of postpartum medical conditions and navigating other time commitments have also proven to be impediments to KMC.^9^

As the benefits of KMC are striking, advocates continue working to overcome implementation challenges. KMC offers a potential solution in resource-constrained settings with high rates of preterm or LBW infants. Malawi is a prime location that could benefit from KMC if implementation can be improved. Reports have estimated preterm birth rates of 18%–26% in Malawi, among the highest globally.^15^^–^^18^ Additionally, prematurity is responsible for over a third of neonatal deaths nationally.^19^^–^^21^

Malawi was an early adopter of KMC, having first introduced it in 1999 and since expanded it to health facilities across the country.^22^ While KMC has been available for the last 2 decades and was included in national policy in 2005, research has noted low levels of post-discharge adherence.^23^ In 2017, KMC was initiated with only 22% of expected cases and 44% of reported preterm or LBW neonates in Malawi.^24^^,^^25^

It is likely that social norms contribute to these low levels of uptake and continuation, as research within Malawi has identified striking sociocultural barriers to KMC engagement.^11^^,^^26^^–^^28^ In a multicountry analysis of bottlenecks to KMC scale-up, stakeholders from Malawi identified community ownership and partnership as their major challenges.^29^ Stakeholders at the Istanbul KMC Acceleration Meeting in 2013 agreed that social and cultural norms are substantial barriers to KMC adoption that must be addressed to accelerate progress.^2^ Hodgins et al. caution the international community to avoid “empty scale-up” of KMC, emphasizing that establishment of KMC units does not necessarily lead to increased coverage of the intervention.^30^ Expanding upon this thinking, effective scale-up of KMC requires a comprehensive approach that understands and addresses social norms.

Effective scale-up of KMC requires a comprehensive approach that understands and addresses social norms that contribute to low levels of KMC uptake.

In 2015, Save the Children conducted research to inform the design, development, and implementation of a pilot KMC social and behavior change communication program in 2 districts in Malawi. The formative research aimed to better understand social norms and community perceptions of preterm infants and KMC to guide program design. Recognizing that KMC requires extensive caregiver involvement, and that caregiver involvement is moderated by social norms, the program centered on social and behavior change communication. We describe this formative research and an analysis of the social norms affecting KMC (facility-initiated and community-continued) in Malawi. Further, we offer an example of a model to link social norms analysis to program implementation to guide efforts in other contexts.

## METHODS

### Study Setting

This qualitative study took place in the districts of Machinga and Thyolo in the Southern Region of Malawi. The 2 districts differ in terms of their sociocultural contexts: Machinga is largely comprised of members of the Yao tribe, with a predominantly Muslim population, whereas most residents of Thyolo are from the Lomwe tribe and identify as Christian. The neonatal mortality rate is similar across both sites (28 per 1,000 live births in Machinga and 27 per 1,000 live births in Thyolo) and on par with the national average of 27 deaths per 1,000 live births.^31^ Across the Southern Region, 54% of newborns receive a postnatal check within 2 days of birth, comparable to the national average of 60%.^31^

Machinga and Thyolo were selected as study sites to inform the pilot program taking place in this area. They were chosen for implementation as they had high birth rates, well-established KMC services to ensure that there would be quality supply to meet increased demand, and ongoing community-based maternal and newborn health initiatives.

In Malawi, health facilities initiate KMC as soon as possible after birth, typically in the KMC ward, with information and support from health providers. Once postpartum KMC parents and their newborns meet facility discharge criteria, they are sent home to continue practicing continuous KMC and requested to return to the health facility for regular follow-up visits until the infant reaches 2,500g, thereby graduating from KMC per national guidelines.^32^ Continuation of KMC at home may be necessary for several weeks after discharge for very preterm infants and is essential to ensure their health. Community health workers, called health surveillance assistants, are trained to follow up with families implementing KMC through household visits and offer continued KMC support.

### Data Collection

Focus group discussions (FGDs) and in-depth interviews (IDIs) were conducted during March–April 2015 with actors across the spectrum of KMC adoption, including pregnant women, parents already engaged in KMC (caregivers), health workers, community members, and religious leaders. Participants were identified through purposive and snowball sampling with assistance from health workers and community leaders. The establishment of FGDs and the number of IDIs was based on purposive and convenience sampling. There was approximately even representation from each district across participant categories.

Interviews were conducted in Chichewa and audio-recorded. FGDs were facilitated with 11 groups of approximately 12 participants each, lasting on average 75 minutes (Table 1). There were 20 IDIs completed, each taking 30–45 minutes. Questions explored participants’ perception of children born before the expected time of delivery and their awareness and perceptions of KMC. Those who had practiced KMC were also asked about their experiences of facility-initiated and community-continued KMC, the challenges they encountered, and recommendations to improve KMC and related services.

**TABLE 1.** KMC Study Participants From 2 Districts in Malawi, by Category

**Participant Category**	**Sample Size, No.**	
Focus group discussions	Individuals	Groups
Pregnant women	48	4
KMC mothers	36	3
Community members	48	4
Total	132	11
In-depth interviews	Individuals	
KMC fathers	6	
Religious leaders	3	
Community leaders	4	
Nurses	3	
Health surveillance assistants	4	
Total	20	

Abbreviations: KMC, kangaroo mother care.

### Ethics Approval

All participants provided informed consent before participating in the interviews. This study was submitted to both the National Health Sciences Research Council in Malawi and the Johns Hopkins Medical Institutions’ Institutional Review Board and was granted exemption from review as it was considered non-human subjects research.

### Data Analysis

Audio recordings were transcribed and translated into English. Analysis followed an inductive thematic approach, whereby codes were developed through an iterative process. Our initial reading of the transcripts detected broad themes, with an immersive re-reading identifying additional codes until a coding structure emerged that a single analyst then applied to the transcripts. Matrices were used to identify patterns across participant type. The Malawi-based research team validated the results and provided contextual interpretation of the findings. Findings from this study focusing on the dissemination of KMC knowledge and skills have been published elsewhere.^33^ The results we describe in this article center on an analysis of the social norms affecting KMC practice.

We were guided by Chung and Rimal’s work explaining how individual behavior is influenced by sociocultural context and related norms, including descriptive norms, injunctive norms, and outcome expectations.^34^ Chung and Rimal define descriptive norms as people’s perception of the prevalence of a behavior in their social environment. They explain that an injunctive norm refers to people’s understanding of what others expect them to do in a social context. Outcome expectations may be positive or negative, denoting social rewards or sanctions conferred in response to a behavior.

## RESULTS

Participants were mainly subsistence farmers who had attended primary school. Most KMC mothers and pregnant women were married, ranging in age from 17–39 years old. KMC fathers (17–60 years) and community members (25–60 years) were on average older. The majority of religious and community leaders were married men. Religious leaders each represented a different religion and/or denomination.

The primary KMC caregivers in this study all identified as the child’s mother, and all described themselves as being in a married relationship with a male partner. As such, the terms she/her/mother/wife and he/his/father/husband are used throughout the results; however, we acknowledge that the primary KMC caregiver, their partner, or support team can be of any gender.

The findings describe the interconnections between community members and KMC parents and highlight the ways in which these relationships affect KMC uptake and continuation. In particular, the data show that social interactions modify KMC parents’ interest in and commitment to KMC, as well as their ability to manage the demands of the practice. As such, the findings are organized through a socioecological model that illustrates the key roles of each group involved in KMC engagement and the related social norms affecting that engagement (Figure). This model is not meant to be exhaustive, but rather to reflect the social norms we identified in this setting and how they affect KMC behavior. In particular, our research focuses on how these social norms operate at the community, family, and household levels.

**FIGURE:**
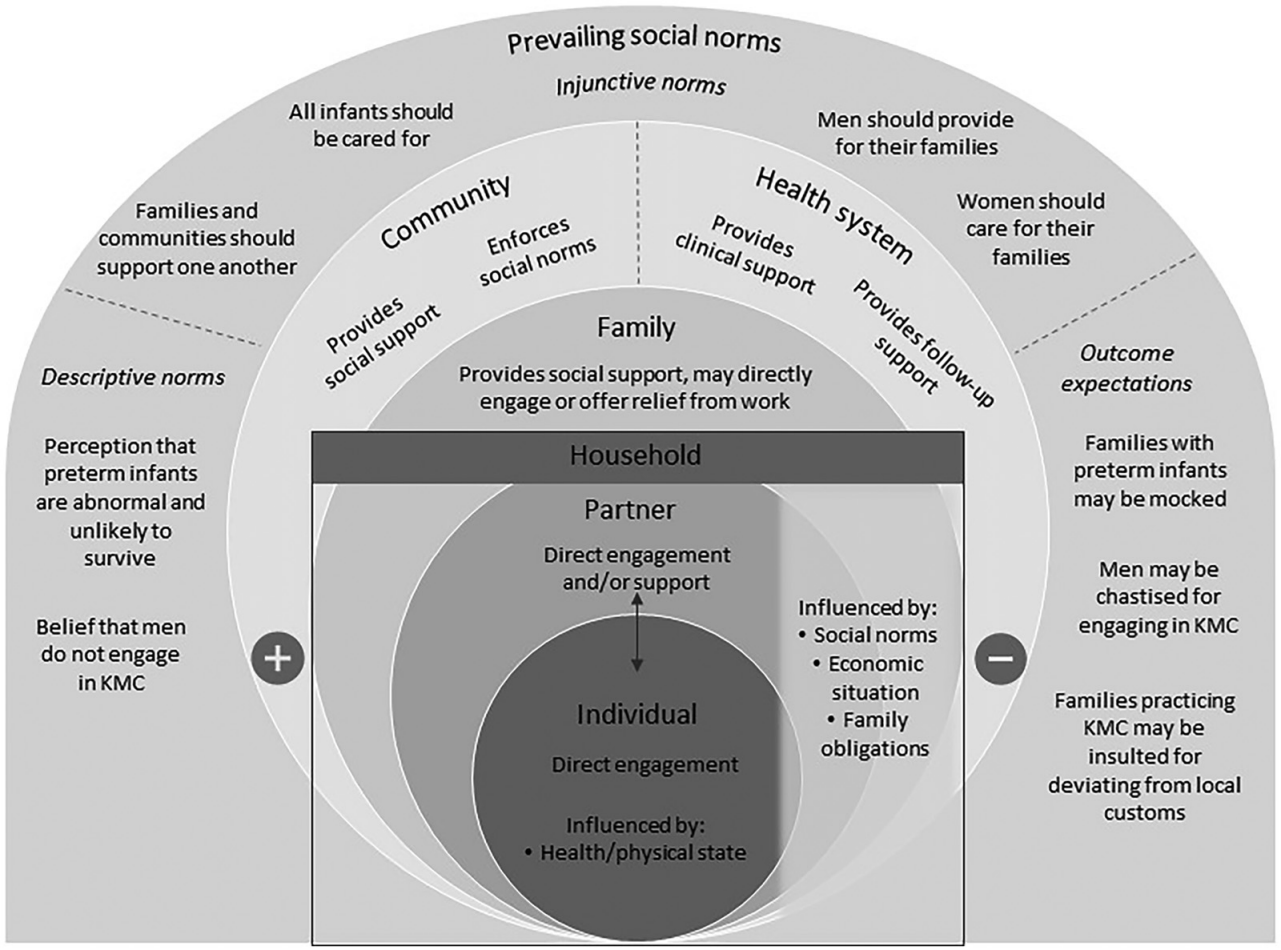
A Socioecological Model of KMC Engagement in 2 Districts in Malawi Abbreviations: KMC, kangaroo mother care.

The data show that social interactions modify KMC parents’ interest in and commitment to KMC, as well as their ability to manage the demands of the practice.

From our analysis, the following 3 main injunctive norms arose: (1) although preterm infants are abnormal, they should still be cared for; (2) men should provide for their families, while women should care for their families; and (3) families and communities should support one another.

These norms, which reflected participants’ perceptions and understood social expectations, were discussed across participant type and cascaded down through the community, affecting family, household, and individual behavior related to KMC.

### Community Level

Community relationships were those with a family’s neighbors and members of their religious community. Community members assumed 2 main roles regarding KMC, as sources of social support and enforcers of social norms.

#### Although Preterm Infants Are Abnormal, They Should Still Be Cared for

KMC parents were asked about their neighbors’ reactions to their preterm infant, and pregnant women were asked about their neighbors’ anticipated perceptions should they deliver preterm. Respondents largely agreed that neighbors held negative attitudes toward preterm newborns. Many of the KMC mothers described being mocked by other women in their community.

*We were also insulted by others who said, “Look at them, they have given birth to an abnormal baby that will not even survive.”* —KMC mother

Many neighbors compared preterm infants to small cats, insinuating that their survival was unlikely and that associating with them was unpleasant.

*Others came around saying, “Those babies in [the KMC ward] are cats and they will die. We don’t even know that these babies will come out [of the hospital].”* —KMC mother

Through these interactions, community members perpetuated the norm that preterm infants are aberrations.

Through interactions with parents, community members perpetuated the norm that preterm infants are aberrations.

The faith community was another important avenue through which norms about preterm infants were developed and reinforced. The Christian and Islamic faith leaders who were interviewed provided insight on the religious understanding of preterm birth. Leaders across both religions tended to describe preterm birth with a negative outlook.

*Our religion laments premature births very much because we understand that if God instituted pregnancies, he meant for the babies to be born after the full term so that they might fulfill God’s purpose for their lives. —*Islamic religious leader

*For me as a Christian leader, I know that when a baby is born prematurely it is an unfortunate situation. Because as leaders, we wonder if perhaps we have failed in our duty to teach people or if we have wronged God. —*Christian religious leader

The religious rhetoric portrayed preterm infants as a deviation from the norm, potentially arising as the result of punishment from God. In line with this, some pregnant women expressed their desire for a full-term infant through religious practice.

*The most important thing is to pray to God so that one day you should be able to deliver a normal child.* —Female community member

At the same time, participants explained how religion teaches the value of life. This belief enforced the norm that even if the community perceives preterm infants as abnormal, they must be cared for.

*They do encourage their flocks not to kill or throw the child away because if you do, you are excommunicated from the church.* —Female community member

*Religion says that if a woman has delivered a preterm baby, she should love that baby*. —Community leader

#### Men Should Provide for Their Families, While Women Should Care for Their Families

Participants described the gendered beliefs held by neighbors about family roles and responsibilities. Community members remarked that the primary role of a father should be to provide economically for his family and disparaged those who did not prioritize this function. Many respondents noted that because their neighbors expected preterm infants to die, they perceived caring for a preterm newborn as an economic burden. This led neighbors to question a father’s competence to appropriately provide for his family when supporting a preterm infant.

*Others would say that her husband is just wasting resources; that is not even a baby.* —KMC mother

#### Families and Communities Should Support One Another

Participants spoke about both their neighborhoods and faith-based communities as sources of social support and as enforcers of the norm that communities should care for their members. It seemed to be routine for religious community members to support families with newborns; additional care and concern were afforded to those with preterm infants. Many described how members of their faith community would visit and share prayers for the family following the birth of a preterm newborn.

Participants spoke about both their neighborhoods and faith-based communities as sources of social support and as enforcers of the norm that communities should care for their members.

*If it happens and your congregation knows about it, they encourage you to take good care of the baby, and even after you are discharged from [the] kangaroo [ward], they come to encourage you, tell you that it happens, and that you must continue caring for the child.* —Pregnant woman

Neighbors also wished to express their care and welcome to the newborn; however, at times, traditional practices conflicted with KMC. In Malawi, it is customary for visitors to hold a newborn when making a house call. As such, women described the tension between practicing KMC properly and abiding by this custom:

*They would insult us, saying we just kept the baby wrapped up and did not hand him/her to them, so we must be snobbish. They did not know that we were protecting the baby’s life.* —KMC mother

The lack of awareness about KMC practices instilled negative attitudes among neighbors. However, several women described neighbors who encouraged KMC and helped them remain hopeful about their child’s condition. These were mainly neighbors who had firsthand experience with preterm infants or KMC:

*If some in the village have been through it before, they might come to advise and encourage you*. —Pregnant woman

### Family Level

The roles and reactions of the extended family were governed by all 3 social norms. KMC mothers and fathers described their relatives having a high degree of concern for the health and well-being of the child. They noted that while some family members initially felt uncertain about the preterm infant, they were receptive to health worker counseling about preterm infants and KMC, leading them to become encouraging advocates. Several respondents explained that their family members knew other women who had preterm infants and witnessed the child’s normal growth and development. This prior exposure prompted family members to support KMC practices and reassure KMC parents that their child can grow up “like any other child.” One participant recounted the support she received from her family:

*As for me, they told me not to get disappointed because I was not the first one to deliver the child before the expected time of delivery. They told me that there are a lot of people who passed the same things, and they are normal now and I would not believe it if I saw them now.* —KMC mother

In particular, female family members adopted a supportive role. Nearly all women described receiving assistance from their female family members, most typically their mothers or mothers-in-law, as well as sisters or cousins. The roles of female relatives were extensive and included performing household chores, supplying the material to wrap the child to the chest (wrappers), and physically engaging in KMC themselves. A strong sense of support emerged through the women’s accounts of their experiences. Here one woman shares what her mother said to her upon learning about KMC:

*You are going to carry your child on your stomach as well, but I am going to help you when you are tired because in here we do help each other, and when I am tired your husband’s mother is going to help me [by] carrying the child on her stomach as well and so on and so forth.* —KMC mother

### Household Level

At the household level, the main relationship described by participants was between the KMC mother and her partner. Community members avidly discussed the role of men in caring for preterm infants, portraying male partner behavior as highly susceptible to gender norms. Many felt that men would not participate in KMC because they would prioritize work, prefer to play games (bawo), or be chastised by their peers.

*Their friends will be saying that he has now become stupid and the wife is the one who gives orders at their home.* —Community member

*If the man plays a part, they say he has been given a love potion.* —Community member

While a few women who had practiced KMC felt that their husbands did not adequately participate in the care of their preterm infants, the majority spoke very favorably about their partners’ involvement. Many women noted that they would physically share in the KMC process, especially at night. Others explained that their husbands would help keep track of the breastfeeding schedule, while some noted that their partners would accompany them to health appointments.

*Whenever I got tired, my husband helped by attaching the baby to his chest, and it went so smoothly that I thought a kangaroo baby is not even difficult.* —KMC mother

When KMC fathers described their involvement in the care of their preterm infants, they emphasized providing material goods for their wives and newborns, including baby clothes, wrappers, and food. Once probed, the men also acknowledged that they physically participated in KMC, especially at night; however, they seemed to value their role as “the provider” more highly. For example, when asked how he cared for his preterm infant, a KMC father responded that as the head of the house, he provided all the things the family needed. Only after several probes to identify all the roles he played did he also note he engaged in KMC.

*I put my child on skin-to-skin care even during sleep time. I always do the kangaroo and sleep whilst my child is still on my stomach*. —KMC father

Many KMC fathers actively practiced KMC; however, they confessed that they felt conflicted by the need to both work and participate in KMC. When engaging in KMC, they grappled with the challenge of maintaining employment outside of the home given the time commitment required by the practice, signaling an economic burden related to KMC.

*The only problem which I found to be difficult was that I was also supposed to put the child on the stomach and this made me not to be able to go for work. This was very difficult for me.* —Male community member

Many KMC fathers actively practiced KMC; however, they confessed that they felt conflicted by the need to both work and participate in KMC.

### Individual Level

KMC mother behavior was driven by a need to care for the family and modified by individual-level characteristics. While women wished to engage in KMC, they were limited by their physical state, available resources, and the need to care for the rest of their families.

In particular, a major challenge expressed by KMC mothers was the physical burden of the practice while they faced their own health challenges. After childbirth, many of the women experienced health complications that made KMC more strenuous, including weakness or pain arising from infection, blood loss, or cesarean birth.

*I had severe abdominal pain after delivery so that I could not attach the baby.* —KMC mother

Even women who did not experience a health complication agreed that KMC is generally quite tiring and painful.

*Another thing is you also get tired, more especially feeling pain on the neck.* —KMC mother

Specifically, women widely acknowledged that practicing KMC while sleeping posed major difficulties.

*It was hard and painful to sleep in one position, which is on our backs.* —KMC mother

KMC mothers were also constrained in their ability to effectively perform KMC by a lack of resources. KMC mothers and the health workers observing KMC families both noted that financial difficulties were common, hindering procurement of enough wrappers and clothing to maintain thermal care for preterm infants. In terms of breastfeeding, a few women noted that they had trouble producing milk; as this is a component of KMC, they felt unable to properly adhere to the practice.

Another key challenge described by KMC mothers was its interference with work. When women first deliver a preterm newborn, they are invited to remain in the KMC ward at the hospital until their infant weighs 2,000g. This process can take several weeks; during this time, the mothers remain at the hospital. This became a concern for some women, as they were unable to contribute to their household during this period.

*When you have to be on kangaroo, the work at home is affected and you find a lot of workload waiting for you since you have to be at the hospital maybe 3 to 4 weeks.* —KMC mother

Participants noted that once home, they are responsible for chores around the house, which are difficult to accomplish without removing the infant from the KMC position.

*If I am all alone at home it can make KMC difficult since I have to work, like to go to the farm, fetch some firewood, and draw water. It is not possible for me to put the child on skin-to-skin always since I have to feed my family as well.* —Female community member

KMC mothers felt conflicted about their need to care for their preterm infant while also performing their household tasks and caring for the rest of their family. These challenges were exacerbated for KMC mothers in specific situations, such as those who gave birth to twins and those without a partner present. Participants experiencing these situations expressed a need for additional support to be able to continue KMC.

KMC mothers felt conflicted about their need to care for their preterm infant while also performing their household tasks and caring for the rest of their family.

### Mapping Social Norms to KMC Implementation

The social norms identified in our results provide insights for KMC efforts. In Table 2, we highlight key findings linked to each social norm, with implications for implementation to underscore areas for intervention. We also provide suggestions for potential corresponding activities to conceptualize ways to integrate these learnings into KMC programs.

**TABLE 2.** Areas for KMC Programmatic Intervention in Malawi

**Social Norm**	**Insight for Implementation**	**Possible Actions**
1. Although preterm infants are abnormal, they should still be cared for.	Build upon the existing shared value for life, including the lives of preterm infants.	Partner with faith and community gatekeepers or influencers to help reinforce message.
	Work to shift community attitudes about preterm infants towards positive perceptions.	Implement a community awareness and advocacy campaign to increase the value of preterm infants and awareness of the effectiveness of KMC. Highlight examples of preterm infants who have grown up to lead fruitful lives and contribute to family life.
2. Men should provide for their families, while women should care for their families.	Highlight the discrepancy between perceived and actual male behavior regarding KMC practices to normalize male involvement.	Engage male champions; popularize male involvement in KMC through interactive community dramas, radio shows, or other mechanisms; reward male KMC engagement in the public sphere.
	Share strategies that support and encourage both KMC parents to engage in the practice collaboratively.	Create a network of KMC alumni parents to share experiences with new KMC parents; impart learnings about KMC role-sharing in initial KMC counseling and ongoing household visits.
	Develop or strengthen efforts to provide economic relief to KMC parents.	Strengthen financial management training or messaging offered with antenatal care to prepare families for the costs related to childbirth, possible complications, and extended hospital stays. Offer families income-generating opportunities in the KMC ward and/or shorten hospital stays and supplement with community follow-up; consider disbursements to KMC families to offset economic losses and/or income-generating activities that can be accomplished while the infant is in the KMC position.
3. Families and communities should support one another.	Encourage existing family and community support systems and link vulnerable families to additional support.	Collaborate with community health workers, local leaders, and Community Action Groups to identify new KMC families that may need additional social support (such as families of twins or those where partner is absent) and link them to formal networks; establish peer mentoring among experienced and new KMC families.
	Strengthen linkages between the health facility and community-based care to ensure continued KMC support at the community level.	Provide an enabling environment for community health workers to support their ongoing KMC follow-up visits (e.g., ensuring transport for household visits, materials, supervision, regular salary). Strengthen the counter-referral system (referral back to the community level from the health facility) to allow for timely community follow-up of KMC families post-discharge.

Abbreviations: KMC, kangaroo mother care.

## DISCUSSION

Our analysis identified 3 injunctive norms affecting KMC engagement in Malawi: (1) a perception that although preterm infants are abnormal, they should still be cared for; (2) an understanding that men should provide for their families while women should care for their families; and that (3) families and communities should support one another. Social norms were shown to influence behavior at all levels of the socioecological model, having both positive and negative effects on KMC practices. In particular, the 3 injunctive norms highlight how care and support are central to the social fabric of Malawi, while also creating conflicting realities for KMC parents. While the parents of preterm infants and the families and communities that surround them are motivated to work together as a family and community unit, they must come to terms with their own perceptions of preterm infants as abnormal and unpleasant. In practice, parents experienced the strain of these conflicting ideas.

The 3 injunctive norms highlight how care and support are central to the social fabric of Malawi, while also creating conflicting realities for KMC parents.

Our findings underscored that KMC is an intensive and arduous practice. It is physically demanding and requires a substantial time commitment. Many previous studies have observed these same challenges, noting a need for behavior change among primary caregivers as well as sufficient social support to overcome such barriers.^9^^,^^12^^,^^14^^,^^26^ The results from this study agree that social support and behavior change are essential for KMC success; however, this study also highlights the critical need for economic support. Parents face competing priorities that underpin the time-commitment barrier described so often. There is a major economic burden associated with KMC through the opportunity cost of lost or reduced work both in and out of the home, affecting the work of both the KMC mother and father. KMC efforts have tended to focus on low-income communities that include subsistence farmers, day laborers, and self-employed vendors, such as the participants in this study. These families simply cannot afford to miss work. The inability to tend the land, procure water and wood, or earn wages while engaging in KMC means families will not be able to meet their basic needs. Further, parents must care for their other children as well, requiring time and energy. While KMC is a low-cost alternative for the health system in comparison to incubator care, the financial burden is shifted to the families engaged in this practice who now must procure wrappers, reduce their work hours, and potentially pay for household help. KMC uptake and adherence will likely remain low unless programs respond to the economic needs of KMC families.

Our results emphasize the important effect of gender norms on KMC engagement. Many participants benefited from a strong female support system among extended family and community members. Unfortunately, this support was often delayed or disrupted by a lack of understanding about preterm infants and KMC practices, also described through a communication lens in previously published work from this study.^33^ Broader family and community sensitization could help prevent gaps in social support and bridge understanding when KMC conflicts with local customs.

Gender norms also influenced community perception, individual identity, and role allocation of KMC parents. Interestingly, while community members did not believe men would engage in skin-to-skin care and detailed numerous social sanctions related to this behavior, nearly all KMC mothers reported the involvement of their husbands. Many parents explained that KMC fathers would attach their child at night when they were home from work. For this reason, community members may not be aware of this male involvement. This discrepancy between the community’s perception of fathers’ behavior and their actual behavior may be critical to expanding KMC engagement in the community. If the community becomes aware of existing male involvement in KMC, this may normalize the behavior and foster increased participation.

Another remarkable facet of the gender norms identified in this context was participants’ internalization of these norms. Men seemed to identify more strongly with their role as the financial and material provider for the family despite also playing a caregiver role. Their engagement in KMC at night and within the confines of the house may be in avoidance of social sanctions, such as being chastised, that would arise if community members were aware of this break in norms. At the same time, this timing of KMC involvement may simply be in response to other work commitments outside of the home during the day. Nevertheless, this role-sharing was greatly appreciated by KMC mothers, as it offered them relief while sleeping. Sharing this strategy with other KMC parents may offer an example of how to collaboratively engage in KMC that can support the needs of both partners—providing KMC mothers with a break from skin-to-skin care at a critical moment and allowing KMC fathers to maintain their work commitments. To help normalize male engagement in KMC, it may also be beneficial to frame KMC as part of their role as a provider for the family, such that provision of thermal care meets the essential needs of their child.

Based on our findings, in Malawi, successful efforts to promote KMC will (1) emphasize the value of life and work to shift community attitudes about preterm infants toward positive perceptions, (2) encourage family and community support systems, (3) highlight the discrepancy between perceived and actual male behavior regarding KMC practices to normalize male involvement, (4) share strategies that support and encourage both KMC parents to engage in the practice collaboratively, and (5) develop or strengthen efforts to provide economic relief to KMC parents.

### An Update on KMC in Malawi Since 2015

With the results of this formative research, the Ministry of Health and Save the Children went on to develop and pilot a social and behavior change communication campaign entitled Khanda ndi Mphatso (“A Baby is a Gift”) in the districts of Machinga and Thyolo. The campaign aimed to reach pregnant women and mothers of preterm and LBW infants, as well as their influencer groups. It included mass media, facility-based activities, and community-based activities, with a focus on shifting social norms and emphasizing the value of newborn life. An evaluation of this program showed significant improvements in individual attitudes toward preterm infants and KMC, as well as significant positive shifts in the injunctive norms described in this study.^35^ A multicountry analysis examined the status of 6 strategic areas for KMC scale-up, identifying improvements in Malawi from 2014–2019 such that the country was noted to have fully achieved all 6 conditions.^15^^,^^36^ This demonstrates notable strides related to national policy and guidance; however, implementation progress and quality improvements tend to lag behind such achievements. As such, further efforts are needed to support families of preterm and LBW infants who can benefit from the lifesaving practice of KMC.

An evaluation of a pilot social and behavior change communication campaign based on this formative research showed significant improvements in individual attitudes toward preterm infants and KMC.

### Limitations

Interpretation of the study results should account for some key limitations. First, it is important to note that the KMC fathers who participated in this study were a small sample and may represent men who were more willing to engage in KMC or to engage in a more extensive manner. As such, the finding that most men shared in attaching their infants to their chests should be interpreted with caution. Additional representative research is needed to confirm this finding. Even if this behavior does not reflect male engagement more broadly, our discussions with participants still yielded important insights about the role of gender norms and how to potentially normalize and increase male involvement in KMC. Second, while our research included an array of participant groups, it was not exhaustive, and influencer groups such as the mothers of KMC parents were not explicitly interviewed. We recommend that future research ask KMC parents who most significantly influences their KMC engagement to be sure to include all relevant influencer groups. Third, it is essential to recognize that social norms are specific to populations and communities; therefore, our results are not widely generalizable. At the same time, our findings highlight elements of KMC practice that are universal and can provide lessons to programs in other contexts. In addition, this research provides a model that can be applied in other settings wishing to introduce or scale up KMC, offering insights about how to understand and link social norms to KMC implementation.

## CONCLUSION

Effective KMC implementation must include an understanding of social norms to tailor implementation to its specific context. Because KMC requires significant caregiver involvement, behavior change that is guided by a socioecological model can prove useful. Successful efforts to increase KMC uptake and continuation will build upon social norms that support KMC and aim to shift social norms limiting the practice, with actions focusing on multiple levels (individual, interpersonal, community, and societal). In contexts where gender roles are very distinct, KMC efforts may be most effective when paired with gender-transformative programming.

## Notes

**Peer Reviewed**

**First published online:** July 27, 2022.
